# A vesicular Na^+^/Ca^2+^ exchanger in coral calcifying cells

**DOI:** 10.1371/journal.pone.0205367

**Published:** 2018-10-31

**Authors:** Megan E. Barron, Angus B. Thies, Jose A. Espinoza, Katie L. Barott, Amro Hamdoun, Martin Tresguerres

**Affiliations:** 1 Scripps Institution of Oceanography, University of California San Diego, La Jolla, CA, United States of America; 2 Department of Biology, University of Pennsylvania, Philadelphia, PA, United States of America; Helmholtz-Zentrum fur Ozeanforschung Kiel, GERMANY

## Abstract

The calcium carbonate skeletons of corals provide the underlying structure of coral reefs; however, the cellular mechanisms responsible for coral calcification remain poorly understood. In osteoblasts from vertebrate animals, a Na^+^/Ca^2+^ exchanger (NCX) present in the plasma membrane transports Ca^2+^ to the site of bone formation. The aims of this study were to establish whether NCX exists in corals and its localization within coral cells, which are essential first steps to investigate its potential involvement in calcification. Data mining identified genes encoding for NCX proteins in multiple coral species, a subset of which were more closely related to NCXs from vertebrates (NCX_A_). We cloned NCX_A_ from *Acropora yongei* (AyNCX_A_), which, unexpectedly, contained a peptide signal that targets proteins to vesicles from the secretory pathway. AyNCX_A_ subcellular localization was confirmed by heterologous expression of fluorescently tagged AyNCX_A_ protein in sea urchin embryos, which localized together with known markers of intracellular vesicles. Finally, immunolabeling of coral tissues with specific antibodies revealed AyNCX_A_ was present throughout coral tissue. AyNCX_A_ was especially abundant in calcifying cells, where it exhibited a subcellular localization pattern consistent with intracellular vesicles. Altogether, our results demonstrate AyNCX_A_ is present in vesicles in coral calcifying cells, where potential functions include intracellular Ca^2+^ homeostasis and Ca^2+^ transport to the growing skeleton as part of an intracellular calcification mechanism.

## Introduction

Coral reef ecosystems are valuable ecological [1] and economic resources [2] centered around the calcium carbonate (CaCO_3_) exoskeletons deposited by scleractinian corals. The aboral ectodermis (also known as the calicoblastic epithelium or calicodermis) is directly above the subcalicoblastic medium (SCM) and the skeleton, and therefore is the tissue layer with the most direct role in calcification ([3]; reviewed in [4]). However, the cellular mechanisms for coral calcification are poorly understood (reviewed in [5]).

Recent research indicates corals exert strong biological control on skeleton formation through intracellular calcification mechanisms. Calicoblastic cells express HCO_3_^-^ transporting proteins that likely supply dissolved inorganic carbon [5–7], as well as coral acidic rich proteins (CARPs) that can catalyze aragonite formation even at pH ~7.6 [8–10]. Furthermore, amorphous CaCO_3_ is present inside coral cells [8] and secreted at the mineralizing front together with HCO_3_^-^, CARPs, and several other proteins [11]. Those results suggest intracellular vesicles play an important role in coral skeleton formation. Another model proposes transcellular Ca^2+^ transport to the skeleton by a combination of Ca^2+^ channels that facilitate Ca^2+^ entry from the coelenteron into the calicoblastic cells [12], and plasma membrane Ca^2+^-ATPases (PMCAs) that extrude Ca^2+^ across the apical membrane into the SCM in exchange for H^+^ (reviewed in [13,14]). However, we have recently reported PMCA is located throughout the cytoplasm of coral calcifying cells and not in the apical membrane, a pattern consistent with localization in intracellular vesicles [7]. Apical Na^+^/Ca^2+^ exchangers (NCXs, SLC8A gene family) have also been proposed to secrete Ca^2+^ from coral calcifying cells into the SCM for skeleton formation [15,16]. However, this model is largely based on osteoblasts from vertebrate animals where NCXs located in the cell plasma membrane mediate bone formation [17,18] and direct evidence for the presence and localization of NCXs in coral cells is lacking.

The goals of the current study were to establish if coral indeed have a protein homologous to the NCXs from osteoblasts, and characterize its expression throughout coral tissues and its intracellular localization in calcifying cells. This type of basic information is an essential first step for future functional experiments to elucidate coral calcifying mechanisms at the cellular level, and to be able to interpret responses to environmental stress based on “-omics” data.

## Materials and methods

### Corals

Colonies of *A*. *yongei* were obtained from the Birch Aquarium at Scripps Institution of Oceanography (SIO) and maintained in flow through seawater (25°C) and a 12:12 hour light:dark cycle in Hubbs Hall at SIO.

### Cloning of AyNCXs

Total RNA was obtained from *A*. *yongei* as previously described [19]. cDNA was synthesized using SuperScript III Reverse Transcriptase (Invitrogen, Carlsbad, CA, USA) and oligo(dT) primers. RT-PCR was performed using primers designed against untranslated regions of predicted *A*. *digitifera* NCX mRNA sequences [20]: AyNCX_A_: FWD primer 5’-AAGCGACTAACCATGTCCTG-3’, REV primer 5’-CTGCTTAAATAACCAGCCCAAAT-3’. AyNCX_B_ FWD primer 5’- CTTGGCGTTCTAGAGAGGTAAAT-3’, REV primer 5’- AAATAACGCGCAACTTGAGAAA-3’ (35 PCR cycles, anneal temperatures of 66°C and 65°C respectively, 1.5 min extension step, using Phusion High Fidelity polymerase (New England Biolabs, Ipswich, MA, USA). After additional PCR rounds using nested primers to further amplify cDNA, bands were gel-purified (NucleoSpin kit, Macherey-Nagel, Düren, Germany), TOPO-TA cloned into a PCR2.1 vector (Invitrogen), and sequenced. Genbank accession numbers for the AyNCXs are MG182344-5.

### Phylogenetic analysis

Amino acid sequences were aligned using MUSCLE [21], trimmed with GBlocks [22], and a maximum likelihood tree with 500 bootstraps was inferred by RAxML (PROTGAMMA model of rate heterogeneity, WAG substitution model). Prediction of transmembrane helices was performed using TMHMM (http://www.cbs.dtu.dk/services/TMHMM-2.0) [23,24]. Prediction of subcellular localization was performed using TargetP 1.1 (http://www.cbs.dtu.dk/services/TargetP/) [25].

### Sea urchin husbandry and gamete collection

Adult *Strongylocentrotus purpuratus* were collected in San Diego, California, and held in 11°C (± 1°C) in flowing seawater aquaria. Animals were spawned by intra-coelomic injection of 0.55 M KCl. Eggs were collected in filtered seawater, washed twice in 0.22 micron filtered seawater (FSW) and kept at 14°C. Sperm was collected and stored at 4°C as described previously [26].

### mRNA synthesis, storage, and dilution

mRNAs encoding C-mCherry *Sp*-ABCC9, *Sp*-ABCB6 [27] and C-mCerulean LCK were made with the SP6 mMessage mMachine kit (Ambion) according to the manufacturer’s protocol and stocks stored at -80°C as previously described [28]. For co-expression experiments, *Sp-*ABCC9 and *Sp*-ABCB6 mRNA were injected at 500 ng mRNA/μL injection solution and 100 ng/μL C-mCerulean AyNCX_A_, while C-mCerulean LCK and C-mCherry AyNCX_A_ were injected at 50 ng/μL and 100 ng/μL, respectively.

### Sea urchin zygote injections

Unfertilized *S*. *purpuratus* eggs were prepared for injection as previously described [29] stuck to 35 mm petri dishes (Fisher Scientific) coated with 0.25% protamine sulfate and fertilized. One-cell zygotes were then injected at between 2–5% egg volume with the mRNA mixture described above. Ampicillin (Sigma Aldrich, St. Louis, MO) at 100 μg/mL in FSW was added to the injection plate. Embryos were then cultured at 15°C (±1°C) for between 16 and 48 hours.

### Imaging and image processing of sea urchin embryos

Injected *S*. *purpuratus* embryos were mounted on 1.5 coverglass (VWR, Radnor, PA) and imaged on either a Zeiss LSM 700 (Jena, Germany) or a Leica Sp8 (Wetzlar, Germany) confocal microscope (mCherry excitation 567 nm, emission 610 nm; mCerulean excitation 433 nm, emission 475 nm). Images were processed using the FIJI distribution of ImageJ [30]

### Antibodies

Custom polyclonal antibodies were developed in rabbit and affinity purified (GenScript USA, Inc) against the peptide antigen sequence KDEDGKSVLRTGEG, which is present in AyNCX_A_ but absent in AyNCX_B._

### Western blot

Coral tissue was removed from the skeleton and homogenized as previously described [7]. The homogenate was sonicated (3 x 10 second bursts, 30 seconds rest between pulses, on ice), and centrifuged at 500 x g for 15 minutes at 4°C to pellet out *Symbiodinium*. Protein concentration in the crude homogenate was determined using the Bradford assay (Bio-Rad, Hercules, CA, USA). Samples were combined with 4x Laemmli buffer (Bio-Rad) with 10% beta-mercaptoethanol and heated at 70°C for 15 minutes. 20 μg protein/lane were loaded into 10% polyacrylamide SDS-PAGE gels and run at 100V for 75 minutes at 4°C. Proteins were transferred onto PVDF membranes on a TurboBlot machine (Bio-Rad) using the pre-programmed 30 minute “Standard Molecular Weight” protocol. PVDF membranes were incubated in blocking buffer (5% powdered fat-free milk in Tris-Buffered Saline + 0.1% Tween detergent (TBS-T)), on a shaker at room temperature for 1h. PDVF membranes were incubated overnight on a shaker at 4°C with primary antibody (0.151 μg/ml; 1:100 dilution from the stock), primary antibody with 400x excess peptide on a molar base (‘pre-absorption control’), or pre-Immune Serum (0.151 μg/ml) in blocking buffer. PVDF membranes were washed with TBS-T (3 x 10 min) and incubated 1 hour on a shaker at room temperature, with secondary antibody (goat anti-rabbit-HRP (BioRad) 1:10,000 in blocking buffer). After washing in TBS-T (3 x 10 min), bands were developed using ECL Prime Western Blot Detection Kit (GE Healthcare, Chicago, IL, USA) and imaged using a Chemidoc Imaging system (Bio-Rad).

### Immunohistochemistry

Coral fragments were fixed and decalcified as previously described [7,19,31], then tissues were dehydrated and embedded in paraffin wax. Wax blocks were cut into 7 μm sections and placed on glass microscope slides. Tissue sections were rehydrated, blocked for 1 hour in blocking buffer (phosphate buffer solution -PBS- with normal goal serum and keyhole limpet hemocyanin solution) and incubated overnight (4°C) with anti-AyNCX_A_ antibodies (1.51 μg/ml), anti-AyNCX_A_ antibodies pre-adsorbed with excess peptide (4.55 μg/ml pre-adsorbed peptide diluted), pre-immune serum (2.36 μg/ml), and blocking buffer alone (“secondary-only” control). The next day, sections were washed in PBS-T (3 x 5 min) and incubated with secondary antibody (goat anti-rabbit-Alexa Fluor555, Invitrogen) (4 μg/ml; excitation 555 nm, emission 568 nm), 1 hour at room temperature. Tissue sections were then incubated with 1 μg/ml Hoechst to stain DNA (5 min, room temperature), washed in PBS-T (3 x 5 min), mounted, and imaged using a fluorescence microscope (Zeiss AxioObserver Z1 with structured illumination) or confocal microscope (Zeiss LSM 700).

Fixed and decalcified coral samples were also processed for immunohistochemistry on 400 nm cryosections [7,32]. Briefly, tissue samples were washed with 0.15 M glycine/phosphate buffer, embedded in 10% gelatin/phosphate buffer and infused with 2.3 M sucrose/phosphate buffer overnight at 4°C. One cubic millimeter blocks were mounted onto specimen holders and snap frozen in liquid nitrogen. Ultracryomicrotomy was carried out at -100°C on a Leica Ultracut UCT with EM FCS cryoattachment (Leica, Bannockburn, IL, USA) using a Diatome diamond knife (Diatome US, Hatfield, PA, USA). 400 nm sections were picked up with a 1:1 mixture of 2.3 M sucrose and 2% methylcellulose (15cp). Sections were permeabilized in PBS-T for three 10-minute washes, incubated in blocking buffer (2% Normal Goat Serum and 2% Bovine Serum Albumin in PBS) for 1h, and incubated with primary antibodies (NCX: 1.51 μg/mL, NKA: 10 μg/mL) overnight at 4°C. Sections were then spot washed with 25μL wash buffer (0.1% Bovine Serum Albumin in PBS) three times, followed by three additional 5-minute washes. Sections were incubated in Alexa Flour 555 goat anti-rabbit secondary (4 μg/ml) together with Hoechst stain (10 μg/mL) for 45 minutes. Sections were washed three times and then imaged. Peptide pre-absorption controls (7.55 μg/mL peptide) were run in parallel.

### Transmission electron microscopy

Corals were fixed overnight in modified Karnovsky’s fixative (2.5% glutaraldehyde and 2% paraformaldehyde in 0.15 M sodium cacodylate buffer, pH 7.4). Coral fragments were then decalcified and post-fixed in 1% osmium tetroxide in 0.15 M cacodylate buffer for 1 hour and stained en bloc in 2% uranyl acetate for 1 hour. Samples were dehydrated in ethanol, embedded in Durcupan epoxy resin (Sigma-Aldrich, St. Lewis, MO, USA), sectioned at 50 to 60 nm on a Leica UCT ultramicrotome (Leica, Bannockburn, IL, USA), and picked up on Formvar and carbon-coated copper grids. Sections were stained with 2% uranyl acetate for 5 minutes and Sato’s lead stain for 1 minute. Grids were viewed using a JEOL 1200EX II (JEOL, Peabody, MA, USA) transmission electron microscope and photographed using a Gatan digital camera (Gatan, Pleasanton, CA, USA).

## Results

### NCX isoforms are present in multiple coral species

**Fig 1:**
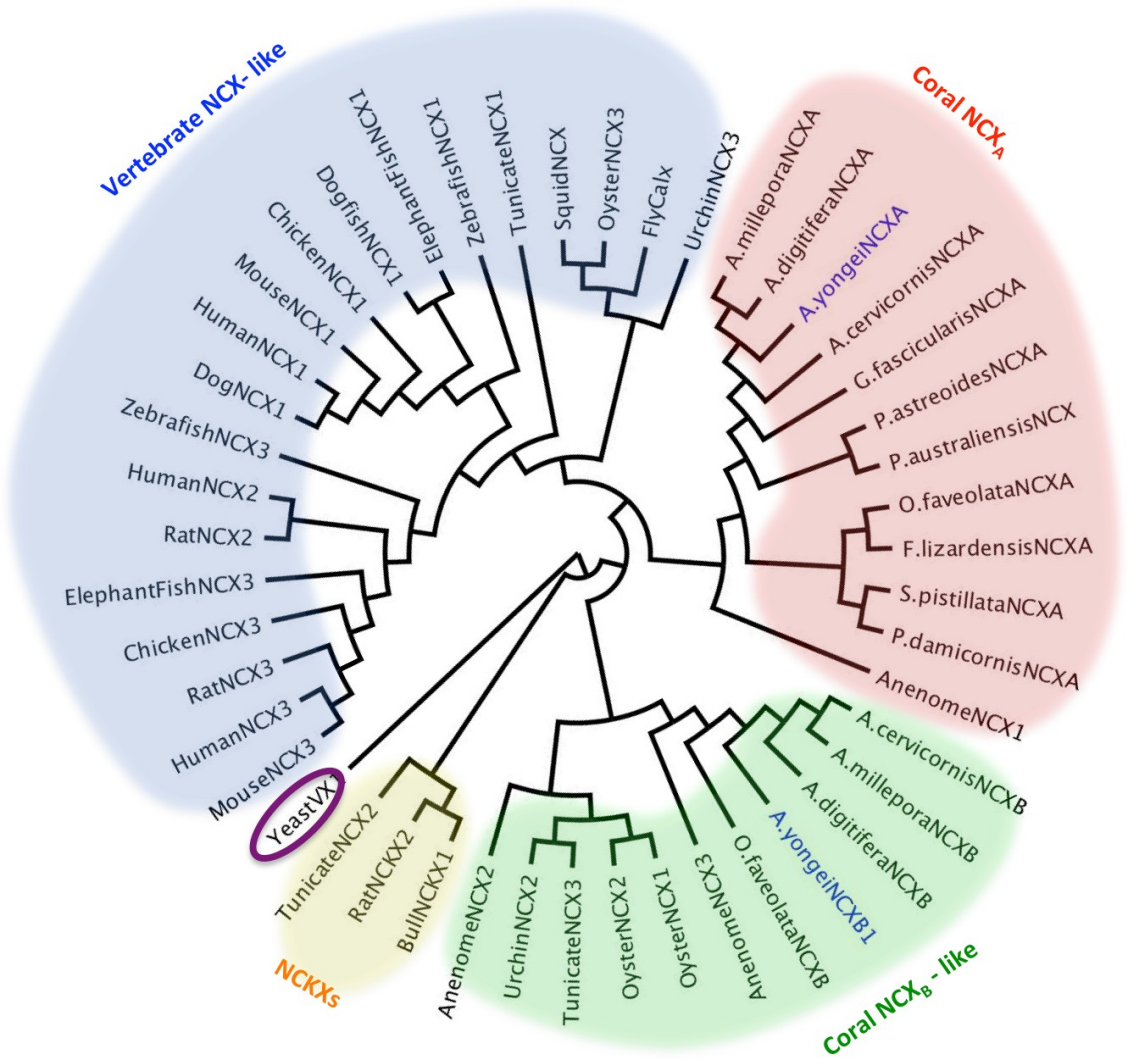
Phylogenetic tree of AyNCX_A_ (MG182344) and AyNCX_B1-4_ (MG182345, MG182346, MG182347, MG182348) (AyNCX sequences are highlighted blue) with other NCX and NCX-like proteins. The following accession numbers were used for obtaining either predicted transcripts, mRNA, or protein sequences. **Coral NCX proteins**: *Acropora digitifera* (A.digitiferaNCXA: XP_015752015.1, A.digitiferaNCXB: XP_015772900.1)^1^, *Acropora millepora* NCX (A.milleporaNCXA: JT007757.1, A.milleporaNCXB: JT003571.1), *Acropora cervicornis* (A.cervicornisNCXA: GASU01080071.1, A.cervicornisNCXB: GASU01087165.1), *Orbicella faveolata* (O.faveolataNCXA: XM_020750226.1, O.faveolataNCXB: XP_020626605.1), *Galaxea fascicularis* (G.fascicularisNCXA: GFAZ01129628), *Porites australiensis* (P.australiensisNCXA: FX462417.1), *Porites astreoides* (P.astreoidesNCXA: GEHP01352486), *Favia lizardensis* (F.lizardensisNCXA: GDZU01041167), *Pocillopora damicornis* (P.damicornisNCXA: GEFF01028265), *Stylophora pistillata* (S.pistillataNCXA: GARY01000181.1 –this sequence was edited to fix frameshift errors that split the protein into three incomplete NCX proteins). All coral sequences were designated A or B based on their homology to AyNCX proteins. **Invertebrate NCX proteins:** *Exaiptasia pallida* (AnenomeNCX1: XP_020915137.1, AnenomeNCX2: XM_021049204.1, AnenomeNCX3: XP_020912295.1), *Strongylocentrotus purpuratus* (UrchinNCX2: XM_011685576.1, UrchinNCX3: XM_011663639.1- this sequence was originally annotated as an NCX1 protein in [36]), *Crassostrea gigas* (OysterNCX1: XP_011444293.1, OysterNCX2: XM_011445979.2, OysterNCX3: XM_020074533.1), *Doryteuthis opalescens* (SquidNCX: AAB52920.1)^2^, *Drosophila melanogaster* (FlyCalx: AAB63464.1)^2^, **Chordate NCX proteins:** *Ciona intestinalis* (TunicateNCX1: XM_002126723.4, TunicateNCX2: XM_002129316.4, TunicateNCX3: XM_002122937.3) *Danio rerio* (ZebrafishNCX1: NM_001037102.1, ZebrafishNCX3: XM_005156997.4), *Callorhinchus milii* (ElephantFishNCX1: XM_007893988.1, ElephantFishNCX3: XM_007893267.1), *Squalus acanthias* (DogfishNCX1: DQ068478.1)^3^, *Gallus* (ChickenNCX1: AJ012579.1, ChickenNCX3: AJ012580.1)^4^, *Rattus norvegicus* (RatNCX2: P48768.1, RatNCX3: P70549.1) ^2^, *Mus musculus* (MouseNCX1: AF004666.1, MouseNCX3: NM_080440.3)^5^, *Canis sp*. (DogNCX1: AAA62766.1) ^2^, *Homo sapiens* (HumanNCX1: NM_021097.2, HumanNCX2: NM_015063.2- the original accession number cited in paper, XM_0038970, no longer exists, HumanNCX3: NM_033262.4)^6^ **NCKX proteins:** *Bos taurus* (BullNCKX1: Q28139.2- was 108825 in reference but number has been updated)^2^, *Rattus norvegicus* (RatNCKX2: AAC19405.1)^2^ **Other:** *Saccharomyces cerevisiae* (YeastVX1: Q99385.1) ^2^, *Homo sapiens* (HumanNCLX: NP_079235.2). NCBI BLAST was used to identify most sequences. Others provided in papers are referenced as follows: ^1^ [20], ^2^ [35], ^3^ [36], ^4^ [37], ^5^ [17], ^6^ [38]. The scale bar represents an amount genetic change of 2.

We cloned two full-length transcripts encoding for putative *A*. *yongei* NCXs (AyNCX_A_ and AyNCX_B_). Similar to NCX proteins from vertebrates (reviewed in [33,34], AyNCX_A_ has a predicted molecular weight of 101.7 kDa and 10 membrane-spanning helices. An alignment of AyNCX_A_ and a mammalian NCX1 is provided in S1 Fig. In addition, AyNCX_A_ contains a peptide signal with very high (0.995) probability to localize the protein to vesicles of the secretory pathway [25]. The other coral NCX that was cloned, AyNCX_B_, has a predicted molecular weight of ~69.8 kDa and only five predicted membrane-spanning helices. We cloned three other cDNAs encoding from putative AyNCX_B_ splice variants with predicted molecular sizes of 26.4, 41.3, and 61.7 kDa. BLAST searches in genomic and transcriptomic databases identified orthologous proteins for both AyNCX_A_ and AyNCX_B_ in multiple other coral species from both the Complex and the Robust clades. Phylogenetic analyses revealed coral NCX_A_ proteins are more closely related to NCXs from vertebrate animals compared to coral NCX_B_ proteins (Fig 1). All these analyses indicate AyNCX_A_ is Na^+^/Ca^2+^ exchanger with ion-transporting properties similar to NCXs from vertebrates. On the other hand, the function of AyNCX_B_ is unclear. Thus, the rest of the experiments focused on AyNCX_A_.

### Recombinant AyNCX_A_ localizes in intracellular vesicles in sea urchin embryos

To further explore the localization of AyNCX_A_ in polarized cells, we took advantage of sea urchin embryos, a recently established heterologous protein expression system for membrane proteins from marine animals [26,27,39]. In this system fertilized embryos (one cell stage) were injected mRNA coding AyNCX_A_ fused to a fluorescent protein and then cultured for the next 16h, during which time the embryo develops a polarized epithelium with apical and basolateral membrane. During this time the exogenous mRNA is translated and the subcellular localization of the corresponding protein, which depends on signal peptides present in the protein of interest, is determined in the embryo by confocal microscopy.

**Fig 2:**
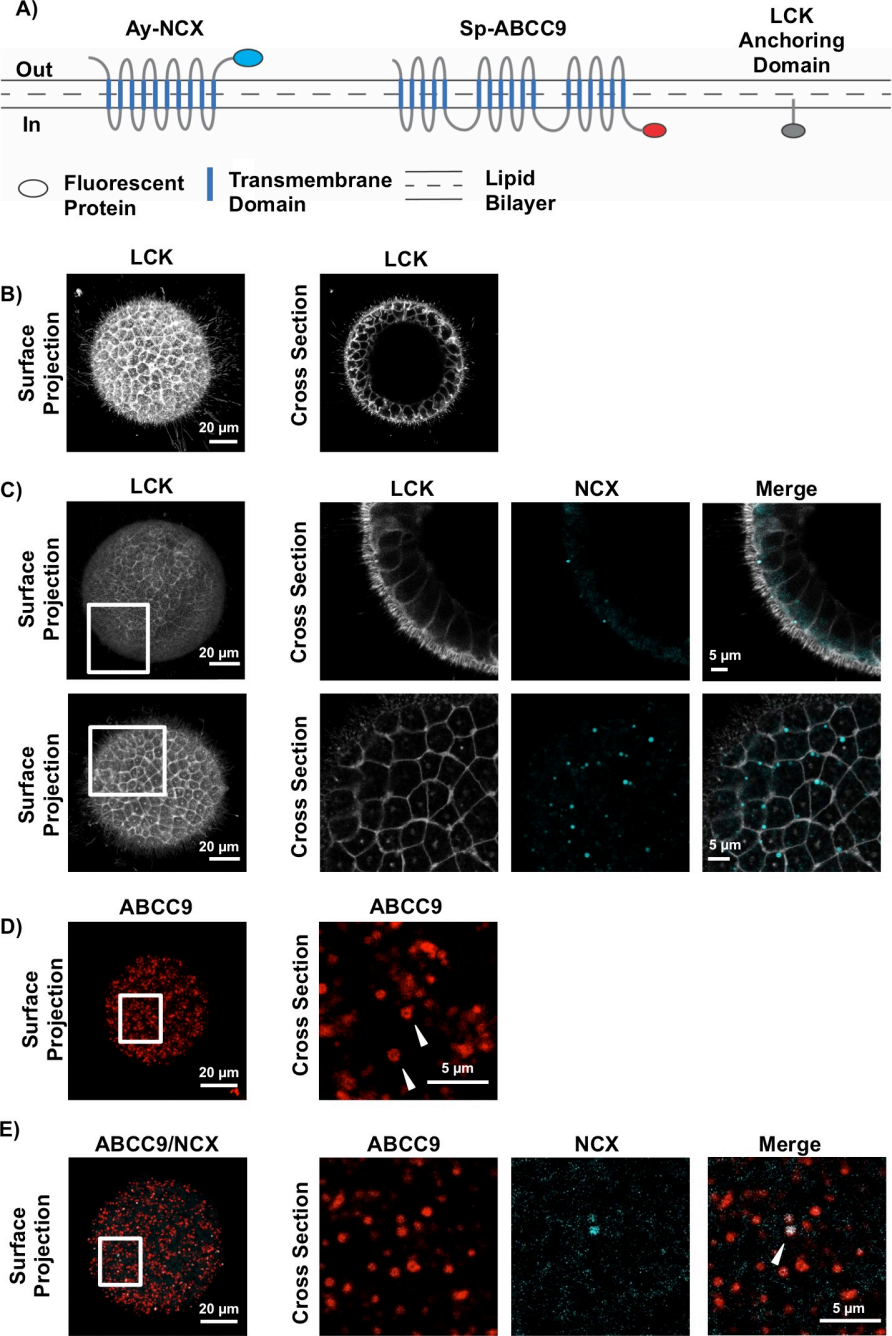
AyNCX_A_ localizes in intracellular vesicles in sea urchin embryos. A) Schematic of the fluorescent protein fusions used in these experiments. Protein colors match the fluorescence in micrographs B-D. B) The sea urchin embryo at ~20 hours post fertilization is a hollow, spherical, epithelial ball approximately 80 μm wide, and LCK is a cell plasma membrane marker. C) Two representative embryos expressing AyNCX_A_ and LCK. Upper row: an equatorial cross section showing AyNCX_A_ vesicles towards the apical surface of the cells. Lower row: Tangential section showing AyNCX_A_ vesicles predominantly at the apical vertices between cells. D) Example of ABCC9 expressing embryo (surface projection) and a zoomed in cross-section with vesicles labeled with white arrows. E) ABCC9 localizes to vesicles, which colocalize with AyNCX_A_ (white arrowhead).

Using this approach, we consistently found that the AyNCX_A_ fusion protein was localized to small spherical intracellular structures ~0.5–1 μm in diameter distributed throughout the cytoplasm. Localization to these structures, presumably vesicles, was regardless of the fluorescent protein to which AyNCX_A_ was fused (mCherry or mCerulean) or the location of the fusion protein (C- or N-terminus) (S2 Fig). Additional controls demonstrated overexpressed mCherry protein is present in the cytoplasm and nucleus but not in vesicles (S3a Fig), and that autofluorescence in uninjected sea urchin embryos is minimal compared to fluorescence from mCherry tagged AyNCX_A_ (S3B Fig). Co-expression of AyNCX_A_ with the plasma membrane marker LCK (Fig 2B) demonstrated AyNCX_A_ is not constitutively present in either the apical or basolateral cell membrane (Fig 2C); however, discreet co-localization events were occasionally observed (Fig 2C) suggesting AyNCX_A_ vesicles might fuse with the cell membrane.

Localization of AyNCX_A_ in mitochondria was ruled out because it did not colocalize with SpABCB6, a mitochondrial protein ([40,41] reviewed in [42]) (S4 Fig). On the other hand, AyNCX_A_ did colocalize with the vesicular protein SpABCC9 (Fig 2E), a sea urchin ATP-binding cassette protein with a readily observable vesicular localization [26]. AyNCX_A_ vesicular localization is consistent with the signal peptide prediction, as well as with the observations on coral calicoblastic cells described below.

### AyNCX_A_ in coral tissue

**Fig 3:**
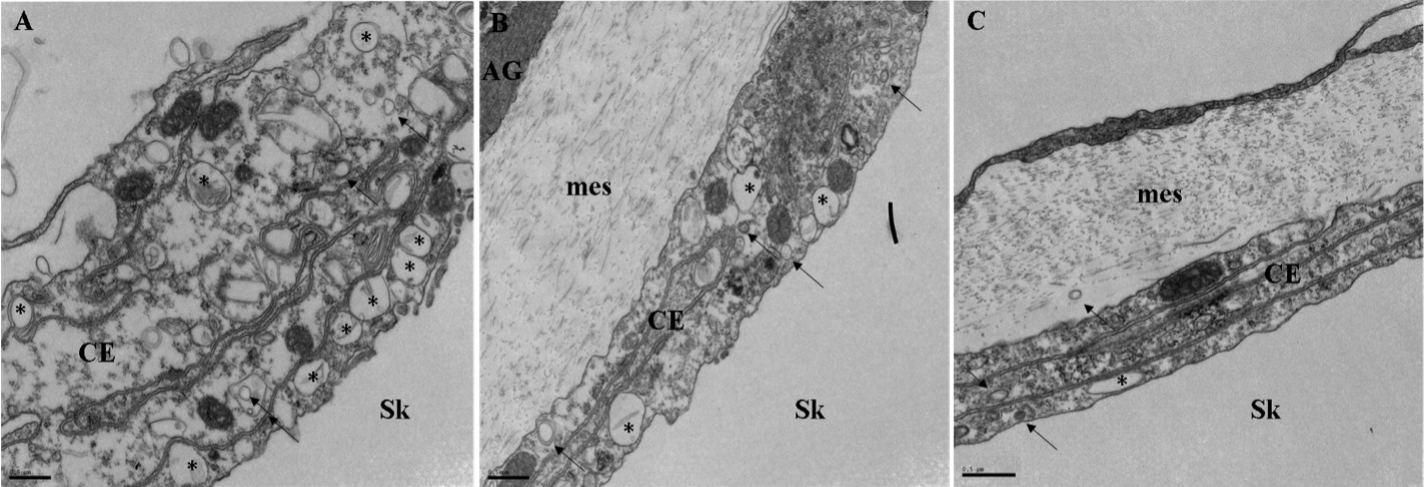
Validation of antibodies against AyNCX_A_. A) The anti-AyNCX_A_ antibodies recognize a ~100 kDa and ~75 kDa protein in homogenized *A*. *yongei* tissue. Both bands are eliminated when the antibody is pre-absorbed with the epitope peptide overnight, and neither band is present when the membrane is incubated with the pre-immune serum. All sample wells contain the same amount of protein and all three Western Blot images were taken at the same exposure. B) Immunofluorescence microscopy of *A*. *yongei* tissue reveals AyNCX_A_ is present in all four tissue layers, including the calicodermis. C) Pre-absorption of antibodies with antigen peptide eliminates signal at the same exposure, confirming antibody specificity.

To determine AyNCX_A_ localization in coral cells, we generated specific antibodies. Western blotting using anti-AyNCX_A_ antibodies specifically recognized two major protein bands in *A*. *yongei* homogenates (Fig 3A): the ~100 kDa band matches the predicted size of AyNCX_A_, and the ~75 kDa band matches the size of a characteristic proteolytic product of mammalian NCX1 [43,44]. Both bands were absent in the peptide pre-absorption and pre-immune serum controls (Fig 3A), validating the specificity of the anti-AyNCX_A_ antibodies.

Immunostaining in 7μm histological sections revealed AyNCX_A_ was present in cells from all tissue layers (Fig 3B). Immunofluorescent signal was absent in peptide pre-absorption controls (Fig 3C) and pre-immune serum controls (not shown), further validating the specificity of anti-AyNCX_A_ antibodies.

**Fig 4:**
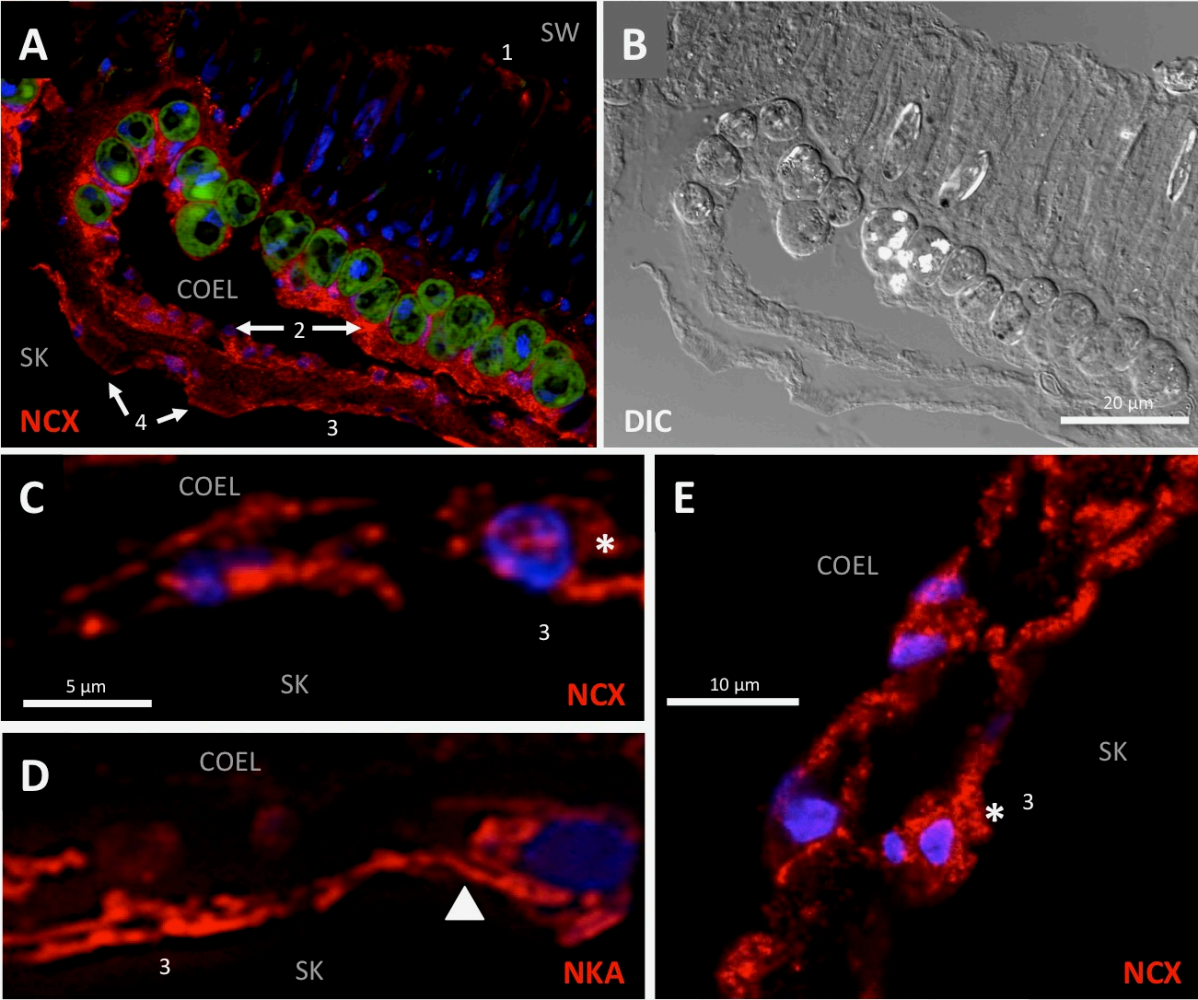
Immunofluorescence microscopy of *A*. *yongei* tissue using structured illumination. A) AyNCX_A_ (red) is present in all four coral tissue layers, including near the apical membrane of the oral ectodermis (labeled 1), and in cytoplasmic structures of oral and aboral gastrodermis (arrows labeled 2), calcifying cells (labeled 3), and desmocytes (arrows labeled 4). B) The corresponding bright field image using differential interference contrast shows cell morphology. C) 400nm-thick cryosection indicates punctate AyNCX_A_ signal in the calicoblastic cells (asterisk). D) 400nm-thick cryosection stained with antibodies against Na^+^/K^+^- ATPase (NKA) provides an example of basolateral staining/localization (arrowhead). E) Confocal microscopy confirms an immunostaining pattern consistent with vesicle localization in calicoblastic cells (asterisk). Nuclei are stained by Hoechst (blue). Abbreviations: SW- Seawater, Coel- Coelenteron, Sk- Skeleton.

**Fig 5:**
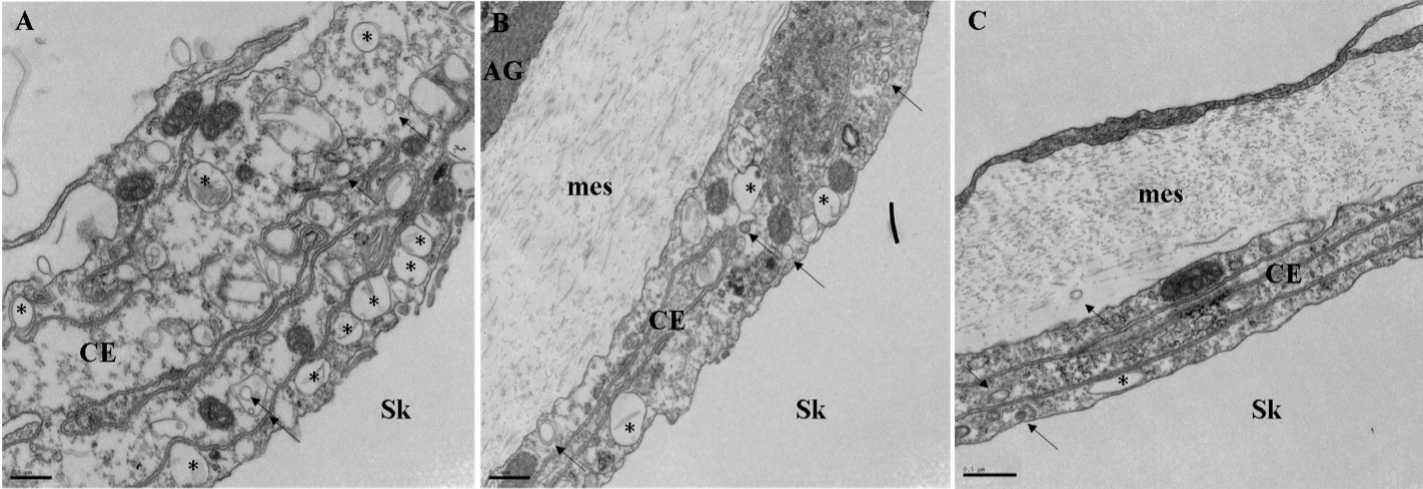
Transmission electron micrographs of the calicoblastic epithelium in *A*. *yongei*. A-C) Vesicles of a variety of sizes are visible in calicoblastic cells. Larger vesicles are indicated by a black asterisk (*), smaller vesicles are indicated by black arrows. Scale bar is 500 nm. Abbreviations: AG- Aboral Gastroderm, mes- mesoglea, CE- Calicoblastic Epithelium, Sk- Skeleton.

Next we looked at AyNCX_A_ subcellular localization in more detail. In the oral ectodermis, AyNCX_A_ was most abundant near the seawater-facing apical membrane of ciliated support cells. In gastrodermal and calicoblastic cells AyNCX_A_ immunostaining pattern was punctate (Fig 4A). Fig 4B shows the corresponding bright field image (differential interference contrast, also known as Nomarski interference contrast). Immunostaining in 400 nm cryosections (Fig 4C) again revealed punctate AyNCX_A_ signal in calicoblastic cells, and clearly different from the basolateral localization of the Na^+^/K^+^-ATPase (Fig 4D) (also compare with Fig 6b in [7]). The punctate AyNCX_A_ immunostaining pattern in calicoblastic cells was also clear in confocal microscopy images (see S1 File, a 3D reconstruction in the Data supplement). In summary, AyNCX_A_ immunofluorescence pattern, bioinformatics analysis, and heterologous expression experiments strongly suggest AyNCX_A_ is present in the highly abundant 80–500 nm vesicles present in coral calicoblastic cells, which are readily visible by TEM (Fig 5).

## Discussion and conclusions

Here we present the first characterization of NCX proteins in coral. We focused on AyNCX_A_, a ~102 kDa protein with 10 predicted transmembrane domains, which is almost identical in structure to the well-characterized NCXs from vertebrate animals and therefore it almost certainly transports Ca^2+^ across cellular membranes in exchange for Na^+^. Orthologous proteins are present in at least nine other coral species from the Complex and Robust coral clades (which diverged from one another over 350 million years ago [45–47]), suggesting NCX_A_ plays a widespread important role in corals.

Unlike NCXs from vertebrate animals that are localized in the basolateral or apical membrane of polarized cells [17,18,37,48,49], AyNCX_A_ is present in intracellular vesicles. This is supported by bioinformatics analyses that revealed a peptide signal typical of proteins present in secretory vesicles, heterologous expression of fluorescently tagged protein in sea urchin embryos demonstrating localization in intracellular vesicles, and immunofluorescence on native coral tissues using specific, custom made antibodies. Interestingly, Na^+^/Ca^2+^ activity is also found in secretory vesicles of mammalian cells [50–52], suggesting vesicular NCXs may also exist in mammals.

Fluorescence microscopy indicates AyNCX_A_ is expressed in all four tissue layers of coral; therefore, NCX_A_ is likely involved in general Ca^2+^ homeostasis processes such as Ca^2+^ sequestration in vesicles. Maintaining a low Ca^2+^ concentration inside cells is essential because Ca^2+^ accumulation in the cytoplasm would precipitate phosphates, interfere with intracellular signaling pathways, and be generally toxic to cells (reviewed in [53]).

Additionally, the relative higher AyNCX_A_ abundance in the calicodermis suggests this Ca^2+^ transporter may be part of a calcification mechanism that relies on intracellular vesicles. Such mechanism used to be favored in early coral research based on the presence of numerous vesicles in the calicoblastic cells of multiple coral species [3,54–57]. Moreover, it was proposed that those vesicles belonged to the Golgi secretory pathway, and that the vesicular membrane regulated Ca^2+^ transport and CaCO_3_ generation [56]. However, those vesicles were not always observed to contain mineralized structures within, and were not always observed fusing with the cell membrane [55]. Although those discrepancies could be at least partially explained by fixation artifacts [55], the model of coral intracellular calcification lost support and was replaced by transcellular Ca^2+^ transport through the cytoplasm of calicoblastic cells (reviewed in [4,14]), paracellular transport of Ca^2+^ regulated by septate junction between calicoblastic cells [58], or bulk transport of seawater to the site of skeleton formation [59]. Importantly, those mechanisms are not mutually exclusive, and all of them involve calicoblastic cells being exposed to high Ca^2+^ levels and therefore the need for robust Ca^2+^ homeostatic regulation.

More recent studies have revived the model for intracellular coral calcification. Amorphous CaCO_3_ was detected as ~400 nm particles throughout *Stylophora pistillata* tissues and then in the skeleton [8], and proteins such as CARPs and carbonic anhydrase were identified in the skeleton matrix and found to have secretory signal peptides [9,10,60,61] (implying exocytosis). In *A*. *yongei*, vesicles in the process of fusing with apical membrane of calicoblastic cells are readily visible by TEM [7], Fig 5). The abundance, size and localization of those vesicles are consistent with the punctate AyNCX_A_ immunostaining pattern, and also matches PMCA’s [7]). Unfortunately, our attempts of immunogold-TEM staining in coral calicoblastic cells have so far been unsuccessful (also see [7]), likely due to the fact that the harsh fixation essential to preserve their complex cellular morphology is not compatible with immunohistochemistry techniques. In fact, to our knowledge this technique has never been performed successfully in coral calicoblastic cells. Nonetheless, the combined evidence indicates AyNCX_A_ is present in vesicles in calicoblastic cells, where it could be regulating intracellular Ca^2+^ homeostasis, participating in skeleton formation, and most likely both.

From an environmental perspective, coral intracellular calcification would confer corals certain resilience to environmental changes in pH and [CO_3_^-^], as recently discussed in detail [8,11]. Thus, vesicular transport of Ca^2+^ and amorphous CaCO_3_ from coral calcifying cells to the skeleton has several important implications. Although the current study suggests NCX_A_ is involved in an intracellular vesicular mechanism for coral calcification, this must be confirmed by functional studies. In this respect, the small size and convoluted morphology of coral calicoblatic cells, together with a lack of tools for studying coral cellular physiology are major limitations (reviewed in [5]). If NCX_A_ was indeed important for coral calcification, it could be used as a biomarker for coral calcification responses to environmental stress; for example by quantifying its mRNA and protein abundance.

## Supporting information

S1 Fig Protein alignment of canine NCX1 (GenBank: P23685.1) and AyNCX_A_ (MG182344.1). Transmembrane (TM) regions annotated on GenBank are highlighted red. Ca^2+^ Binding Domains (CBD1, CBD2) [62] are highlighted yellow and blue, respectively. The protein alignment was made using EMBOSS Needle [63].
(PDF)

S2 Fig Sea urchin embryos expressing coral AyNCX_A_ tagged with fluorescent protein. A-C) AyNCX_A_ with Cerulean Fluorescent Protein (CFP) at the N-terminus. D-F) AyNCX_A_ with CFP at the C-terminus. G-I) AyNCX_A_ with mCherry fluorescent protein at the N-terminus. J-L) AyNCX_A_ with mCherry fluorescent protein at the C-terminus. For each set of 3 images, the left image (A,D,G,J) shows a single z-stack at the base of the embryo, the middle (B,E,H,K) shows a z-stack through the middle of the embryo, and the right (C,F,I,L) is a z-project of all z-stacks. Embryos expressing CFP-tagged AyNCX_A_ were imaged 16hpf, embryos expressing mCherry-tagged AyNCX_A_ were imaged 24hpf.
(TIFF)

S3 Fig Untagged mCherry and uninjected sea urchin controls. A) mCherry lacking an Ay-NCX_A_ or Sp-ABCC9a fusion localizes diffusely in the cytoplasm, and does not localize to intracellular vesicles. B) Quantification of Ay-NCX_A_ mCherry positive intracellular vesicles relative to uninjected negative controls. mCherry-only positive vesicles were counted in Ay-NCX_A_ vs background in negative control embryos. N = 12 embryos. Error bars are +/- SEM, and comparisons were made using Student’s T-Test. Inset: example Ay-NCX_A_ and control embryos.
(TIF)

S4 Fig Sea urchin embryo expressing C-CFP-AyNCX_A_ and C-mCherry-ABCB6, an urchin protein localized in the mitochondria. A-C) a single z-plane from the base of the urchin embryo showing A) CFP-AyNCX_A_, B) mCherry-ABCB6, and C) the two images merged. D-F) a z-project of all z-planes showing D) CFP-AyNCX_A_, E) mCherry-ABCB6, and F) the two images merged. G) The merge, enlarged, shows there is no co-localization of the two proteins (would appear white).
(TIFF)

S1 File 3D reconstruction of coral tissue stained with anti-AyNCX_A_ antibodies (red). Nuclei are indicated by Hoescht dye (blue).
(PPTX)
