# Correction: A vesicular Na^+^/Ca^2+^ exchanger in coral calcifying cells

**DOI:** 10.1371/journal.pone.0209734

**Published:** 2018-12-19

**Authors:** The PLOS ONE Staff

Fig 3 is incorrect. The authors have provided a corrected version here. The publisher apologizes for the error.

**Fig 3:**
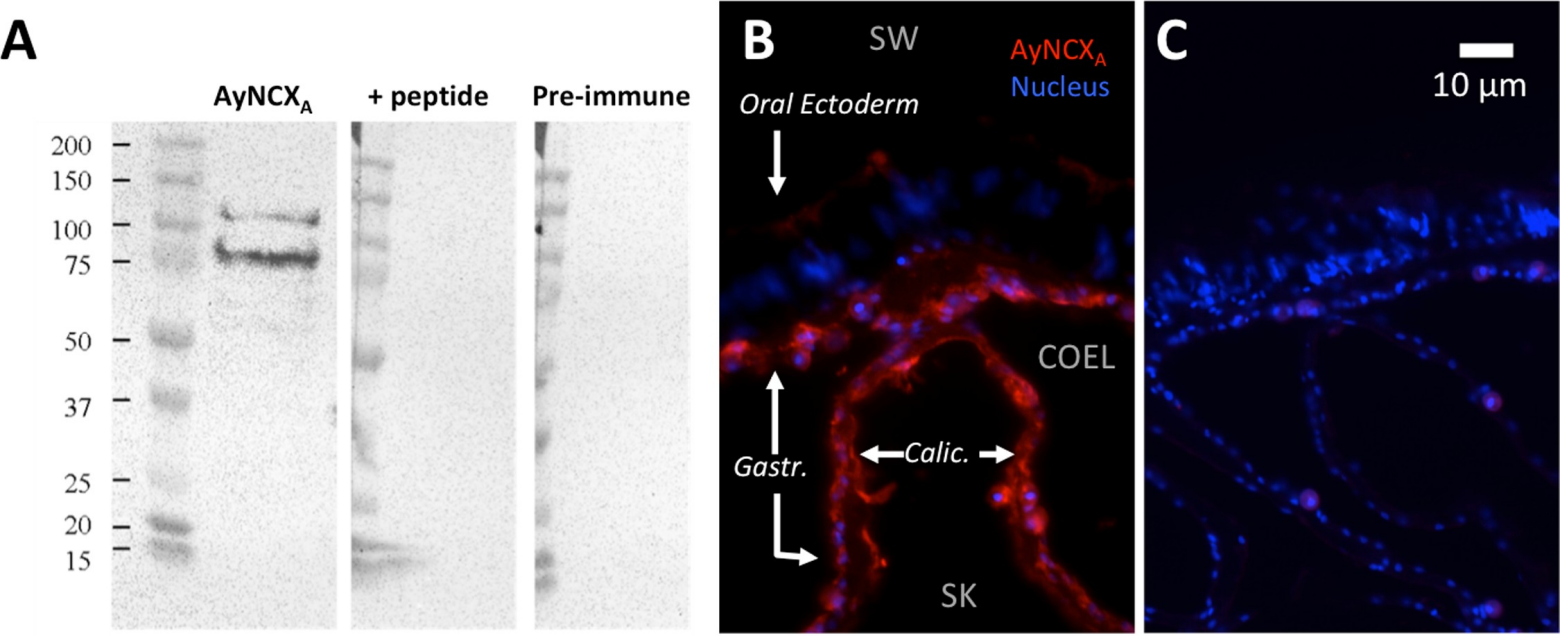
Validation of antibodies against AyNCXA. A) The anti-AyNCX_A_ antibodies recognize a ~100 kDa and ~75 kDa protein in homogenized *A*. *yongei* tissue. Both bands are eliminated when the antibody is pre-absorbed with the epitope peptide overnight, and neither band is present when the membrane is incubated with the pre-immune serum. All sample wells contain the same amount of protein and all three Western Blot images were taken at the same exposure. B) Immunofluorescence microscopy of *A*. *yongei* tissue reveals AyNCX_A_ is present in all four tissue layers, including the calicodermis. C) Pre-absorption of antibodies with antigen peptide eliminates signal at the same exposure, confirming antibody specificity.
