# Supplementary material for: A vesicular Na+/Ca2+ exchanger in coral calcifying cells
Source: PLoS One. 2018 Oct 31;13(10):e0205367. doi: 10.1371/journal.pone.0205367 (PMC6209159; doi:10.1371/journal.pone.0205367)
Supplement: S1 Fig — Transmembrane (TM) regions annotated on GenBank are highlighted red. Ca2+ Binding Domains (CBD1, CBD2) [62] are highlighted yellow and blue, respectively. The protein alignment was made using EMBOSS Needle [63]. (PDF) [file pone.0205367.s001.pdf]

```
CanineNCX1         1 MLQLRLLPTFSMGC--HLLAVVALLFSHVDLISAETEMEGEGNETGECTG     48
                                   |.|   .|:..:..::| :.....:..::...:||  |..
AyNCX1             1 -----------MSCVQRLVLWLGFIWS-IWCTFLQQTVQAADDET--CNL     36
                                                      TM 1
CanineNCX1        49 SYYCKKGVILPIWEPQDPSFGDKIARATVYFVAMVYMFLGVSIIADRFMS     98
                     :.:||:||:||:|  ..:||.|:|:.||.||.:|::|.||||||||:|||||
AyNCX1            37 NNHCKEGIVLPVW-GGNPSTGEKVGRAFVYLLALLYFFLGVSIISDRFMS     85
                                                                   TM 2
CanineNCX1        99 SIEVITSQEKEITI-KKPNGETTKTTVRIWNETVSNLTLMALGSSAPEIL    147
                     :||:|||:||||| .|..|:....||::||||||||||||||||||||
AyNCX1            86 AIEIITSKEKEITITDKQTGKEKIVTVKLWNETVSNLTLMALGSSAPEIL    135
                                                   TM 3
CanineNCX1       148 LSVIEVCGHNFTAGDLGPSTIVGSAAFNMFIIIALCVYVVPDGETRKIKH    197
                     ||.||:||:||.||:|||||||||||||:.||||:||||:|||:.|:|||
AyNCX1           136 LSAIEICGNNFKAGELGPSTIVGSAAFNLLIIIAVCVYVIPDGDVRRIKH    185
                                   TM 4                   TM 5
CanineNCX1       198 LRVFFVTAAWSIFAYTWLYIILSVISPGVVEVWEGLLTFFFFPICVVFAW    247
                     ||||.:||:.|:.||.||||||:|.|...||:||.||||.||||.|:.|:
AyNCX1           186 LRVFAITASTSVLAYVWLYIILAVSSKNEVEIWEALLTFLFFPIMVLAAY    235

CanineNCX1       248 VADRRLLFYKYVVYKRYRAGKQRGMIIEHEGDRPSSKTEIEMDGKVVNSHV    297
                     ||||||||:.:.:|.|..|:.|:.:...||       .::.|..|.
AyNCX1           236 VADRRLLFYRALRRRQRRQKRAGLSVVQTGD-------FDIIGVQVK---    275

CanineNCX1       298 DNFLDGALV-------LEVDERDQDDE------EARREMA-RILKELKQK    333
                     |.|:||.:.        .|..:...|||      |.|||.| :.||||::||
AyNCX1           276 DGFVDGNVADGRHTGDAEAGQDFDDDELLHDLTEDRREKAIQALKEIRQK    325

CanineNCX1       334 HPEKE---IEQLIELANYQVLSQQQKSRAFYRIQATRLMTGAGNILK-RH    379
                     ||..:    :|:|:|..|   |..|.|||||||||:|||.|.|:||:|| :|
AyNCX1           326 HPHADRETVERLLEQEN---LKLQPKSRAFYRIEATRKMVGSGNVLKMKH    372

CanineNCX1       380 AADQARKAVS--------MHEVNTEVAENDP-VSKIFFEQGTYQCLENCG    420
                       |:..||.|          |.::..|    || |.:::|:...|...||||
AyNCX1           373 --DKLEKASSSVADAKLEMQDIEYE----DPQVVRVYFDPVHYIVKENCG    416
                                     Ca2+ Binding Domain 1
CanineNCX1       421 TVALTIIRRGGDLTNTVFVDFRTEDGTANAGSDYEFTEGTVVFKPGETQK    470
                     :|.:.|.|.|||.||||:||||||||||.||.|||.|||||.|:|||||.|
AyNCX1           417 SVFMNITRTGGDPTNTVYVDFRTEDGTAEAGGDYEKTEGTVCFRPGETTK    466
                                                   Overlap of CBDs
CanineNCX1       471 EIRVGIIDDDIFEEDENFLVHLSNVKVSSEASEDG-ILEANHVSALACLG    519
                     ...|.||||||||||||:|.|||.||:|    ||||| :|...:......:|
AyNCX1           467 TFSVVIIDDDIFEEDEHFYVHLGNVRV---ASEDGDLLRNKYRGPAGKVG    513

CanineNCX1       520 SPSTATVTIFDDDHAGIFTFEEPVTHVSESIGIMEVKVLRTSGARGNVIV    569
                     ....||:||.|||:.|||||||....::|:.|.:::||:|.:||||.|.|
AyNCX1           514 KDGVATITILDDDYPGIFTFEEEKYTITEADGTIKLKVVRHTGARGTVRV    563
                                    Ca2+ Binding Domain 2
CanineNCX1       570 PYKTIEGTARGGGEDFEDTCGELEFQNDEIVKTISVKVIDDEEYEKNKTF    619
                     ||.|:||||:|||.|:||..|||||:|||..|||.:.||||||||||:||
AyNCX1           564 PYHTVEGTAKGGGTDYEDAVGELEFENDETWKTIEINVIDDEEYEKNETF    613

CanineNCX1       620 FLEIGEPRLVEMSEKKALLLNELGGFTITGKYLYGQPVFRKVHAREHPIP    669
                     ::.||||::|:..|              .||
AyNCX1           614 YVVIGEPKVVKKDE--------------DGK-------------------    630

CanineNCX1       670 STVITIAEEYDDKQPLTSKEEEERRIAEMGRPILGEHTKLEVIIEESYEF    719
                      :.:...|.||          .|:.|:||||:|.||:.:|.||.|.||.||
AyNCX1           631 -SDLRTGEGYD---------AEKERLAEMGKPRLGDVSKAEVTIIESKEF    670

CanineNCX1       720 KSTVDKLIKKTNLALVVGTNSWREQFIEAITVSAGEDDDDDECGEEKLPS    769
                     |:|||||:.|.||||||||:||:|||::|:|||..:|::..|  |:..|:
AyNCX1           671 KNTVDKLLVKANLALVVGTSSWKEQFVDALTVSGSDDNNGSE--EQSTPT    718
```

```
                                                         TM 6
CanineNCX1      770 CFDYVMHFLTVFWKVLFAFVPPTEYWNGWACFIVSILMIGILTAFIGDLA     819
                    ..||:||::|||||:|||.||||:.|.|||||:||.:.||:||..|||:|
AyNCX1          719 YGDYMMHYMTVFWKLLFAIVPPTDIWGGWACFVVSTVFIGLLTMVIGDVA     768
                                TM 7
CanineNCX1      820 SHFGCTIGLKDSVTAVVFVALGTSVPDTFASKVAATQDQYADASIGNVTG     869
                    |||||||||.|||.|:.|||||||:||||||||||..|:|||:|||||||
AyNCX1          769 SHFGCTIGLADSVVAITFVALGTSLPDTFASKVAAIGDEYADSSIGNVTG     818
                                TM 8                                TM 9
CanineNCX1      870 SNAVNVFLGIGVAWSIAAIYHAANGEQFKVSPGTLAFSVTLFTIFAFINV     919
                    ||:||||||:|||||||||.:.|.||.|.|..|:|.|||.:|.:.|.:.:
AyNCX1          819 SNSVNVFLGLGVAWSIAAIANKAKGENFVVEAGSLGFSVVVFCVCALVAI     868
                                                  TM 10
CanineNCX1      920 GVLLYRRRPEIGGELGGPRTAKLLTSCLFVLLWLLYIFFSSLEAYCHIK-     968
                    .||:.||..::||||||.:..||.||.||.:||::||..|||:.||:|:
AyNCX1          869 AVLMLRRSKKVGGELGGAKPYKLPTSLLFAMLWMIYIVLSSLQTYCYIRP     918

CanineNCX1      969 GF    970
                    ||
AyNCX1          919 GF    920
```
