# Supplementary figures and images for: A vesicular Na+/Ca2+ exchanger in coral calcifying cells
Source: PLoS One. 2018 Oct 31;13(10):e0205367. doi: 10.1371/journal.pone.0205367 (PMC6209159; doi:10.1371/journal.pone.0205367)

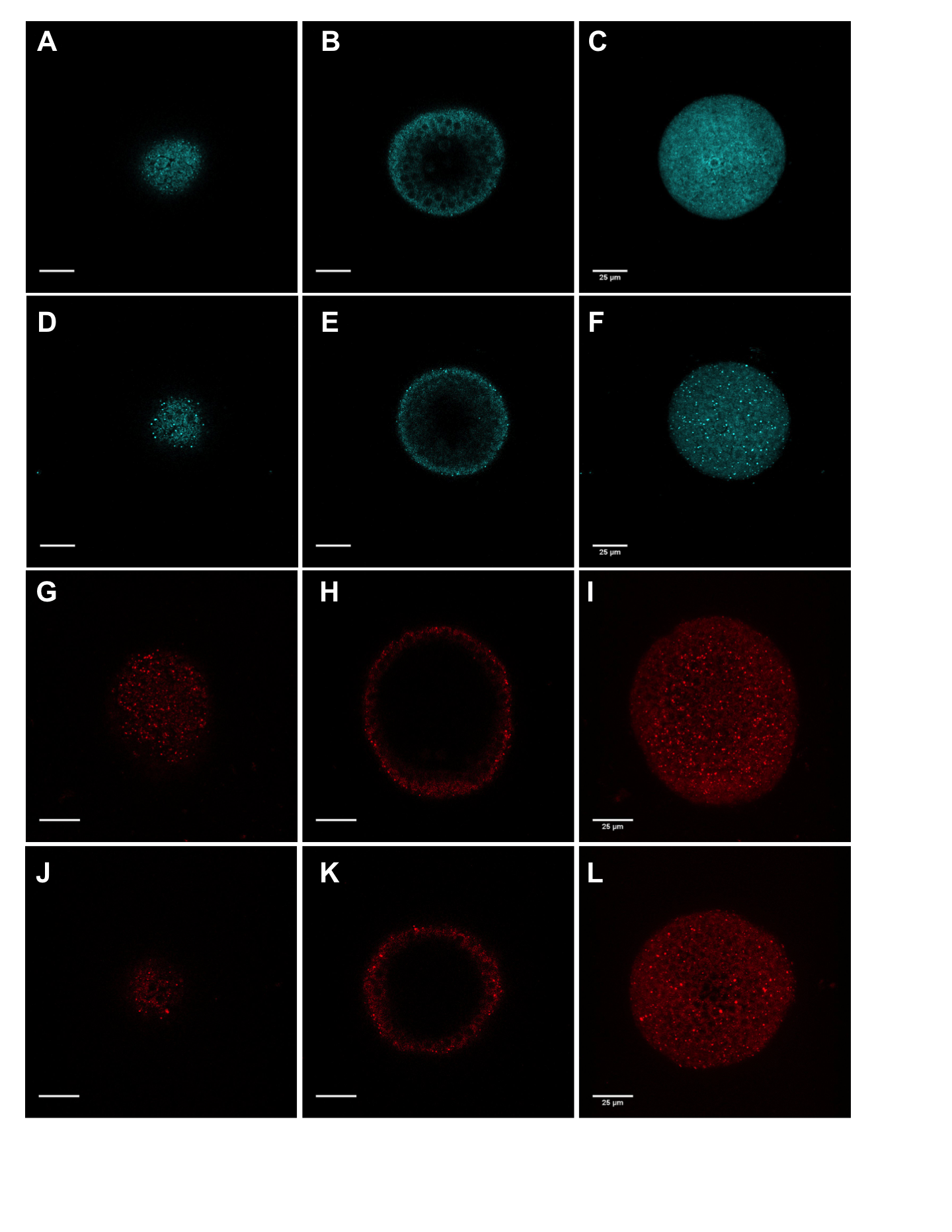

Supplement: S2 Fig — A-C) AyNCXA with Cerulean Fluorescent Protein (CFP) at the N-terminus. D-F) AyNCXA with CFP at the C-terminus. G-I) AyNCXA with mCherry fluorescent protein at the N-terminus. J-L) AyNCXA with mCherry fluorescent protein at the C-terminus. For each set of 3 images, the left image (A,D,G,J) shows a single z-stack at the base of the embryo, the middle (B,E,H,K) shows a z-stack through the middle of the embryo, and the right (C,F,I,L) is a z-project of all z-stacks. Embryos expressing CFP-tagged AyNCXA were imaged 16hpf, embryos expressing mCherry-tagged AyNCXA were imaged 24hpf. (TIFF) [file pone.0205367.s002.tiff]

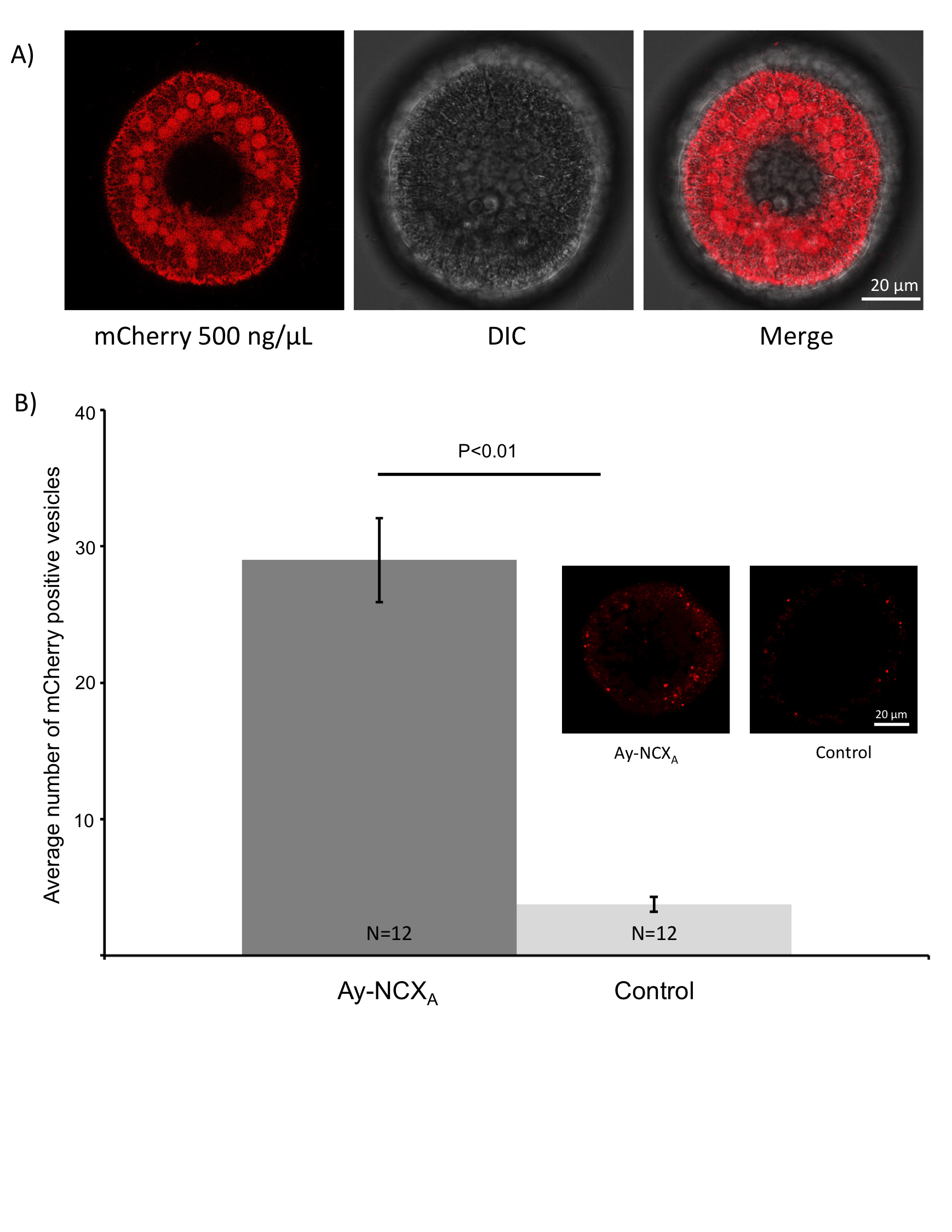

Supplement: S3 Fig — A) mCherry lacking an Ay-NCXA or Sp-ABCC9a fusion localizes diffusely in the cytoplasm, and does not localize to intracellular vesicles. B) Quantification of Ay-NCXA mCherry positive intracellular vesicles relative to uninjected negative controls. mCherry-only positive vesicles were counted in Ay-NCXA vs background in negative control embryos. N = 12 embryos. Error bars are +/- SEM, and comparisons were made using Student’s T-Test. Inset: example Ay-NCXA and control embryos. (TIF) [file pone.0205367.s003.tif]

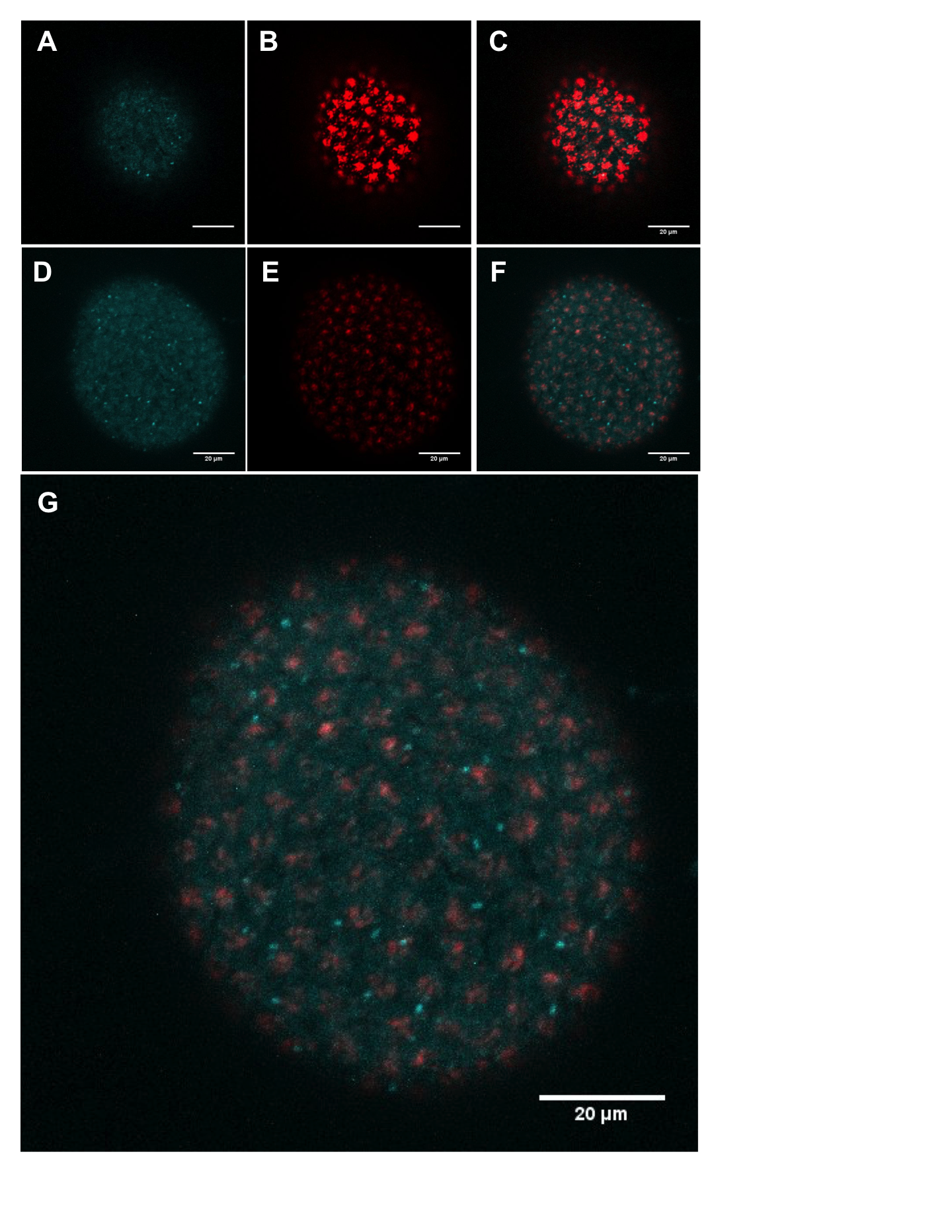

Supplement: S4 Fig — A-C) a single z-plane from the base of the urchin embryo showing A) CFP-AyNCXA, B) mCherry-ABCB6, and C) the two images merged. D-F) a z-project of all z-planes showing D) CFP-AyNCXA, E) mCherry-ABCB6, and F) the two images merged. G) The merge, enlarged, shows there is no co-localization of the two proteins (would appear white). (TIFF) [file pone.0205367.s004.tiff]

## Slide 1
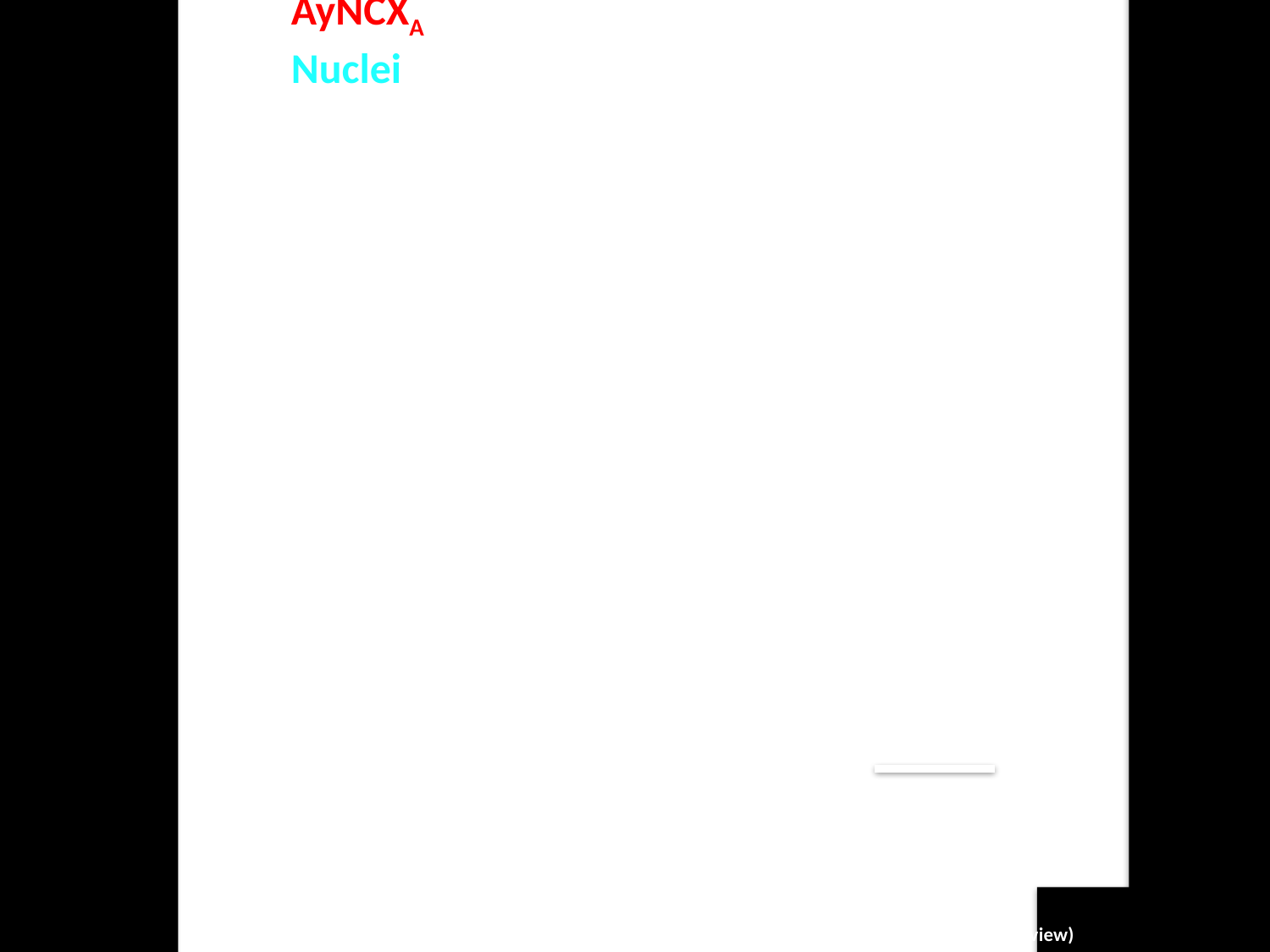

AyNCXA
Nuclei
Coelenteron / Gastroderm
10 μm
Calicodermis
Barron et al (in review)

Supplement: S1 File — Nuclei are indicated by Hoescht dye (blue). (PPTX) [file pone.0205367.s005.pptx]
